# In silico structural modeling and analysis of physicochemical properties of curcumin synthase (CURS1, CURS2, and CURS3) proteins of *Curcuma longa*

**DOI:** 10.1186/s43141-020-00041-x

**Published:** 2020-07-02

**Authors:** R. Santhoshkumar, A. Yusuf

**Affiliations:** Interuniversity Centre for Plant Biotechnology, Department of Botany, University of Calicut, Malappuram, Kerala 673635 India

**Keywords:** CURS, Curcuminoids, In silico analysis, Bioinformatic tools, Homology modeling

## Abstract

**Background:**

Pharmaceutically important curcuminoid synthesis in *C. longa* is controlled by CURS1, CURS2, and CURS3 genes. The present study detected the physicochemical properties and structural characteristics including the secondary and 3D structure of CURS proteins. The primary, secondary, and tertiary structure of the CURS proteins were modeled and characterized using multiple bioinformatics tools such as ExPasy ProtParam tools, self-optimized prediction method with alignment (SOPMA), PSIPRED, and SWISS-MODEL. The predicted secondary structure of curcumin synthase provided an α-helix and random coil as the major components. The reliability of the modeled structure was confirmed using PROCHECK and QMEAN programs.

**Results:**

The molecular weight of CURS1 is 21093.19 Da, theoretical pI as 4.93, and an aliphatic index of 99.19. Molecular weight of CURS2 and CURS3 proteins are 20266.13 Da and 20629.52 Da, theoretical pI as 5.28 and 4.96, and an aliphatic index of 89.30 and 86.37, respectively. In the predicted secondary structure of CURS proteins, alpha helices and random coils of CURS1, CUR2, and CURS3 were 42.72, 41.38, and 44.74% and 24.87, 31.03, and 17.89, respectively. The extended strands were 16.24, 19.40, and 17.89. QMEAN Z-score is − 0.83, − 0.89, and − 1.09 for CURS1, CURS2, and CURS3, respectively.

**Conclusion:**

Prediction of the 3D model of a protein by in silico analysis is a highly challenging aspect to confirm the NMR or X-ray crystallographic data. This report can contribute to the understanding of the structure, physicochemical properties, structural motifs, and protein-protein interaction of CURS1, CUR2, and CURS3.

## Background

The major class of secondary metabolites from *C. longa* contains a mixture of curcumin (60–80%), demethoxycurcumin (15–30%), and bisdemethoxycurcumin (2–6%) [1], soluble in methanol, ethanol, or dimethyl sulfoxide and insoluble in water [2]. Curcuminoids have anti-inflammatory, antimutagenic, anti-diabetic, anti-bacterial, and hepatoprotective activities [3]. It is also known for its free-radical scavenging antioxidant activity [4], healing of the dermal wound [5], and prevention of Alzheimer’s disease [6]. Most importantly, curcumin inhibits the cell growth of various cancer cell lines and induces apoptosis in cancer cells [7] and also in the regulation of cancer cell growth [8].

Curcumin synthesis is mediated by curcumin synthase, (CURS), the gene family has three members; curcumin synthase 1 (CURS1, the first identified CURS) and type III polyketide synthases (PKSs), *Viz*. CURS2 and CURS3, having CURS-like activity with the substrate specificity slightly different from that of CURS1 [9] involved in curcumin synthesis pathway. Type III polyketide synthases (PKSs) consists of structurally simple homodimers of ketosynthase that are involved in the biosynthesis of most of the plant polyketides [10].

The elucidation of protein structure is one of the key features for understanding the biological processes at a molecular level. However, very little is known about the structure of CURS (CURS1, CURS2, and CURS3) proteins. Identification of the 3D structure of a protein is very difficult and complex. X-ray crystallography or NMR spectroscopy methods were used to determine the protein structure, but it is time-consuming and not successful with all proteins, particularly in membrane proteins [11]. A viable alternative approach developed to predict the in silico 3D structure of proteins based on homology modeling using an unknown protein sequence with more than 35% of similarity [12] serves the purpose with better validation.

The present study was aimed at modeling curcumin synthase genes of *C. longa* using in silico analysis including physicochemical properties of the designed secondary structure, modeling CURS protein 3-D structure, evaluation, and analysis of the modeled structures using different standard computational tools.

## Methods

### Plant material

*Curcuma longa* rhizomes were collected and identified using flora and conserved as field germplasm, a voucher specimen was submitted to the herbarium and a voucher number (6949) was provided by the curator of the herbarium. The rhizomes were harvested after 10 months of cultivation and used for extraction. Analytical grade chemicals purchased from Hi-Media Laboratories, Mumbai, India, were used for the extraction.

### Cloning and annotation of putative CURS gene

Total RNA from *C. longa* rhizome was isolated using modified SDS method [13]. Purified RNA was converted into cDNA using Takara PrimeScript^TM^ RT reagent kit (Cat. # RR037A, Takara Bio Inc., Japan) according to the manufacturer’s instructions. The cDNA synthesis reaction mixture contained 5X Primescript buffer (2.0 μl), Primescript RT enzyme mix (0.5 μl), 0.5 μl Oligo dT primer (50 μM), 2.0 μl random hexamers (100 μM), template RNA (10 μl), and the final volume was adjusted to 20 μl by adding RNase-free water. The reaction conditions were reverse transcription at 37 °C for 15 min, inactivation of reverse transcription at 85 °C for 5 s, and hold at 4 °C. Primers for cloning the CURS gene were designed from the conserved regions of available *C. amada, C. longa,* and *C. zedoaria* CURS genes retrieved from GenBank (Accession Nos. CURS1—KM880189.1 *C. longa* CURS1, AB495007.1 *C. longa* CURS1 and MF402846.1 *C. zedoaria* CURS1; CURS2—KF980981.1 *C. amada* isolate CURS2-XI CURS2, KF980982.1 *C. amada* isolate CURS2-XII CURS2, LC064068.1 *C. longa* CURS2, AB506762.1 *C. longa* CURS2; and CURS3—KX154461.1 *C. amada* CURS3, AB506763.1 *C. longa* CURS3, KM880190.1 *C. longa* CURS3, and MF987835.1 *C. zedoaria* CURS3) using Multalin and Primer-BLAST. The primers designed were CURS1 (F:5′-ATGGTGAAGA AGCGGTACCTG-3′; R: 5′-TGTTGCCGTACTCTGTGAAGA-3′), CURS2 (F:5′-GCTAATC AGTCAATCCAGA TGG-3′; R: 5′- CGTCTATCGATTGATCGATC GT-3′), and CURS3 (F:5′-GTCAACCG CCTCATG CTCTACA-3′; R:5′-TCACCTCGTCCAT CACGAAGTAC-3′). PCR was carried out using 10× PCR buffer (2 μl), 25 mM MgCl_2_ (2 μl), 100 mM dNTPs, forward primer 1 μl, reverse primer1 μl, ~ 50 ng cDNA template, and .25 μl Taq DNA polymerase (5 U/μl) and the final volume was made up to 25 μl with sterile double distilled water. The reaction conditions were initial denaturation at 95 °C for 15 min and 35 cycles comprising: 95 °C for 20 s, gradient annealing temperature at (51.5, 52.1, 53.4, 54.0, 55.4 55.9, 58.6 and 59.6 °C) for 40 s, 72 °C for 1 min, and final extension at 72 °C for 10 min. Amplified PCR products were visualized on a 1% (w/v) agarose gel and molecular weight was detected using standard 1 kb DNA ladder. The PCR product was purified and sequenced. The obtained sequence was analyzed using BLAST (http://www.ncbi.nlm.nih.gov) program to find out the homology of the sequence and submitted in NCBI (MK515083, MG386668, and MK511334) translated to corresponding proteins. The Open Reading Frame (ORF) Finder program was used to determine the coding regions of the sequences and the sequences were annotated.

### Physicochemical characteristics

The physical and chemical attributes, such as molecular weight, theoretical pI, amino acid composition, atomic composition, extinction coefficient, estimated half-life, instability index, aliphatic index, and grand average of hydropathy (GRAVY) of the CURS proteins, were computed using Expasy ProtParam tool [14].

### Secondary structure prediction

The secondary structure properties like the α-helix, β-sheet, and turn of amino acid sequences of CURS proteins were predicted using PSI-blast-based secondary structure PREDiction (PSIPRED) [15] and self-optimized prediction method with alignment (SOPMA) [16].

### Protein 3D model prediction

The derived CURS protein sequences were used as query sequences for comparative modeling. SWISS-MODEL (http://swissmodel.expasy.org) was used for the 3D structure prediction of CURS1, CURS2, and CURS3 and its integrated external resources, such as UniProt, InterPro, STRING, and Nature PSI SBKB were also used for analysi s[17].

### Model evaluation

Different tools were used to evaluate the internal consistency and reliability of the modeled structure of the CURS1, CURS2, and CURS3. PROCHECK and MolProbity programs were used to assess the stereochemical quality of the model by quantifying the residues in the allowed zones of Ramachandran plot [18]. The obtained protein structure was re-assessed for its reliability and model quality using QMEAN Z-scores from QMEAN server http://swissmodel.expasy.org/docs/structure_assessment [19].

## Results

### Cloning and annotation of putative CURS gene

PCR-assisted cloning using the designed primers amplified the CURS1, 900 bp; CURS2, 1100 bp; and CURS3, 590 bp, genes and the homology determination provided similarity with the existing CURS genes from the genebank. The ORF finder demarcated a putative 588 bp, 675 bp, and 570 bp ORF for the three cloned CURS nucleotide sequences translated to CURS proteins with 195, 224, and 190 amino acid residues for CURS1, CURS2, and CURS3 with ATG as the initiation codon.

### Physicochemical properties

Different physicochemical properties of the CURS proteins were examined using ExPASy ProtParam tool (Table 1). The molecular weight of CURS1 is 21093.19 Da, theoretical pI 4.93, and an aliphatic index of 99.19. The instability index was 32.10 and GRAVY was 0.199. Molecular weight of CURS2 and CURS3 proteins are 20266.13 Da and 20629.52 Da, theoretical pI as 5.28 and 4.96, and an aliphatic index of 89.30 and 86.37, respectively. The instability index was 37.84 and 31.33 and GRAVY was 0.118 and 0.058 for CURS2 and CURS3. Phosphorylation sites were predicted using NetPhos 2.0 server. The CURS1 protein has 3 Ser, 2 Thr, and 1 Tyr; CURS2 has 4 Ser, 5 Thr, and 2 Tyr; and CURS3 showed 4 Ser, 1 Thr, and 3 Tyr.

**Table 1:** Physicochemical properties of CURS proteins

S. no.	Name of the proteins	M. wt. (Da)	Seq. length	pI	EC (assuming all pairs of Cys residues form cystine)	EC (assuming all Cys residues are reduced)	Half-life (h)	II	GRAVY	−R	+R	AI
1	CURS1	21093.19	197	4.93	28,085	27,960	30	32.10	0.199	22	14	99.19
2	CURS2	20266.13	224	5.28	21,095	20,970	30	37.84	0.118	20	13	89.30
3	CURS3	28629.52	190	4.95	28,085	27,960	30	31.33	0.058	23	14	86.37

*M. wt.* molecular weight, *pI* isoelectric point. *−R*, number of negative residues, *+R* number of positive residues, *EC* extinction coefficient at 280 nm, *II* instability index, *AI* aliphatic index, *GRAVY* grand average hydropathy

### Secondary structure prediction

**Fig. 1:**
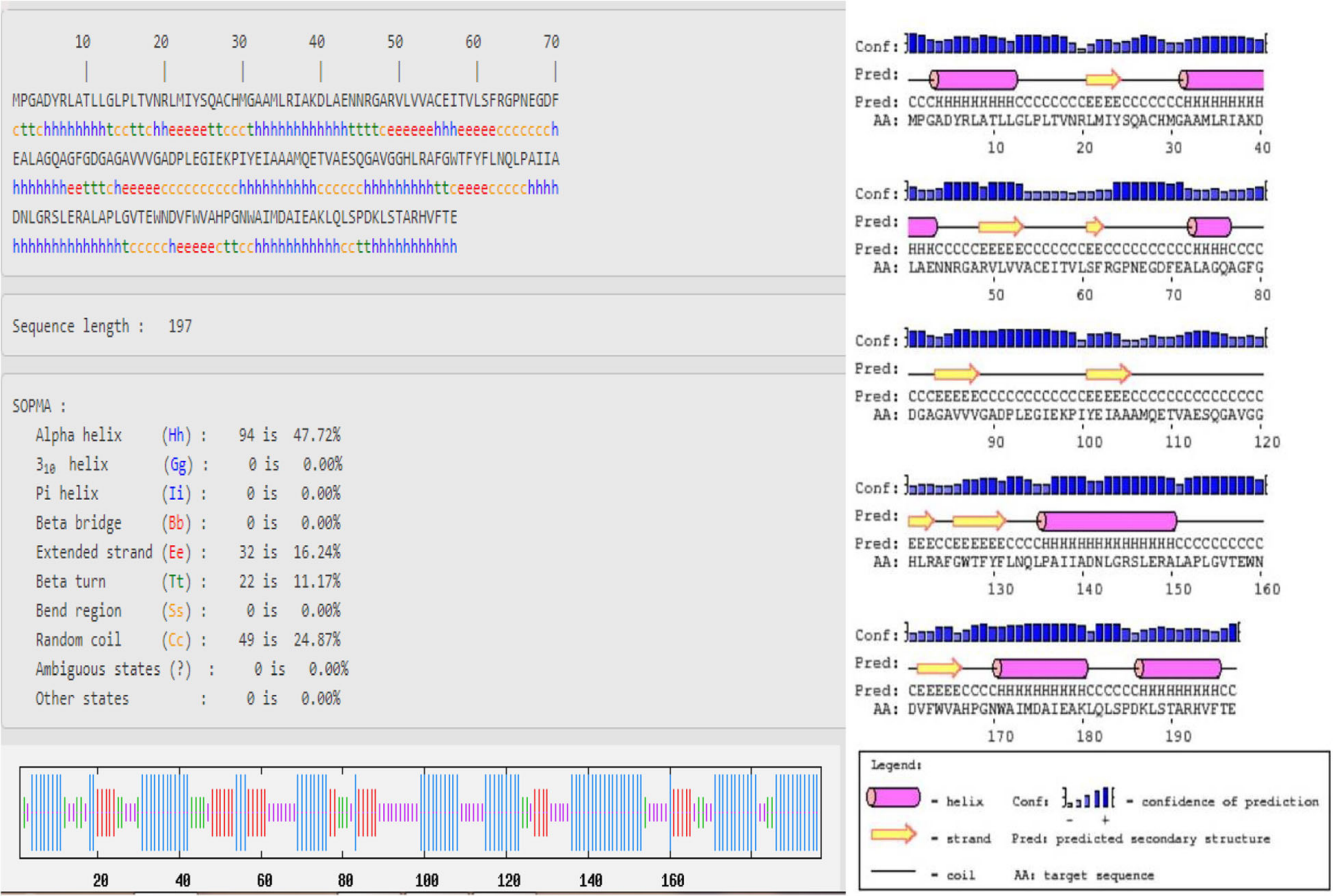
Secondary structure analysis of *C. longa* CURS1

**Fig. 2:**
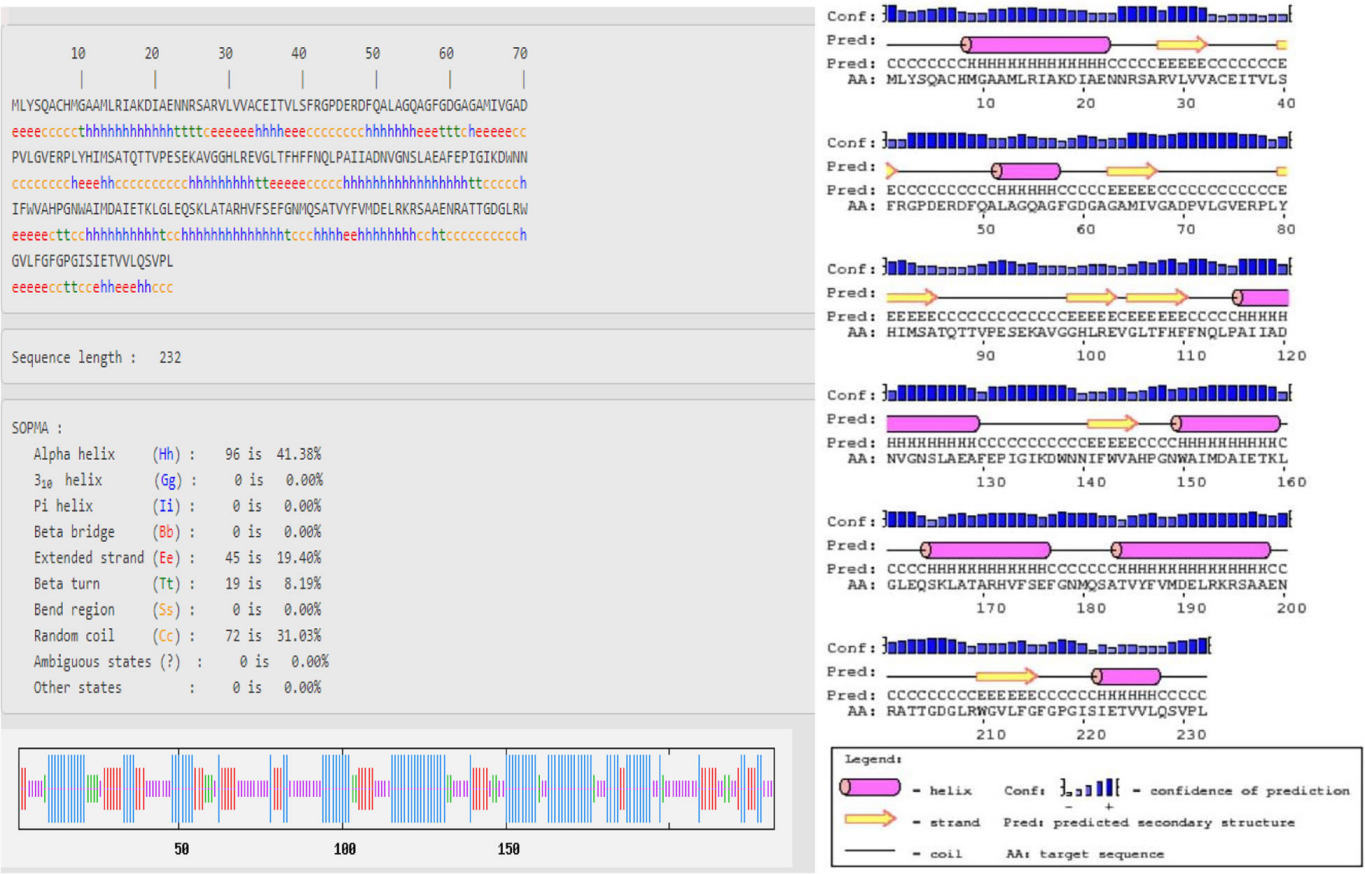
Secondary structure analysis of *C. longa* CURS2

**Fig. 3:**
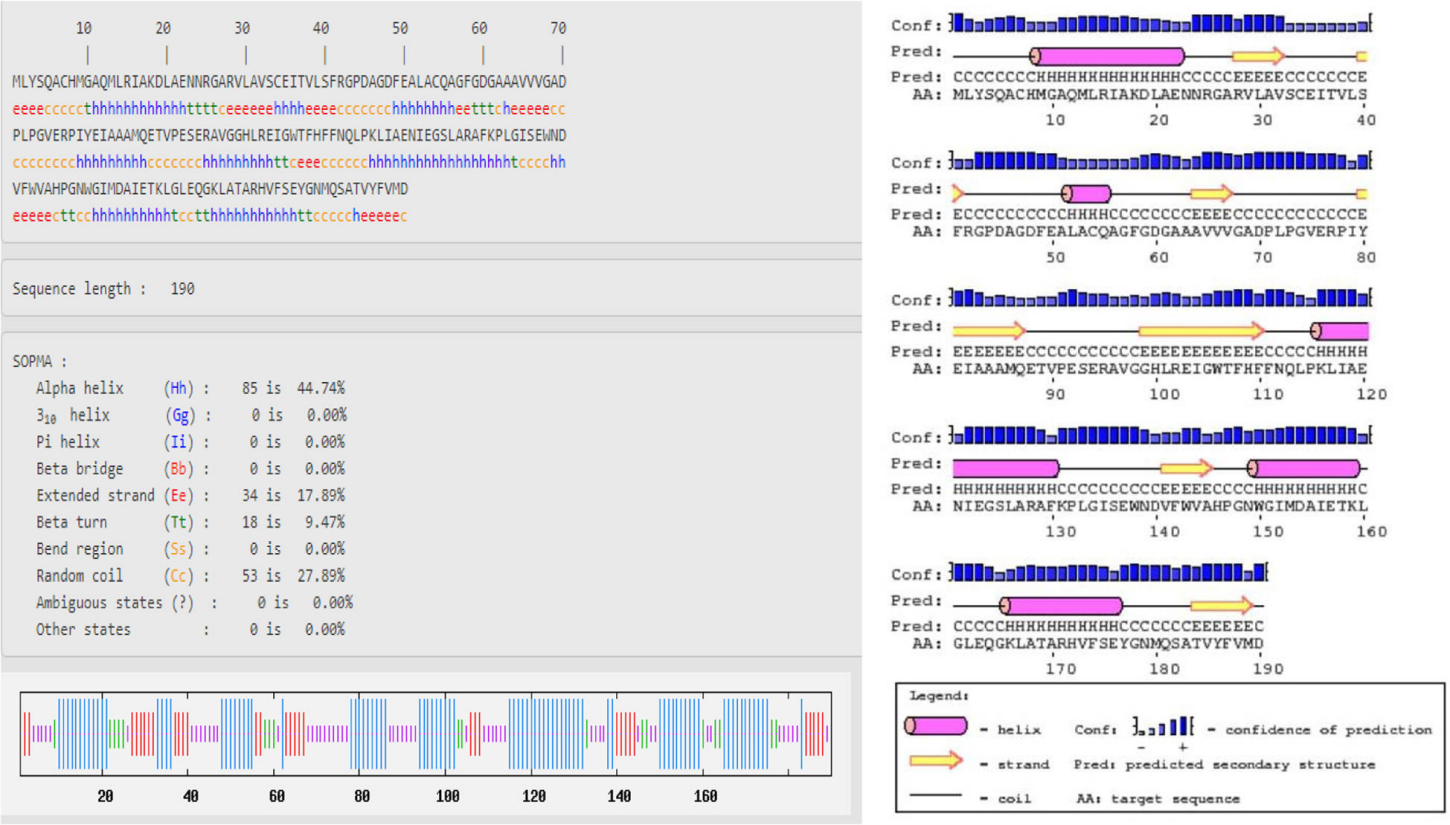
Secondary structure analysis of *C. longa* CURS3

The secondary structure of protein chains was analyzed by SOPMA that predicted the alpha helix, extended strand, beta turn, and random coil (Figs. 1, 2, and 3). In the designed secondary structure of CURS proteins, alpha helices showed 42.72, 41.38, and 44.74% in CURS1, CUR2, and CURS3, respectively. It is followed by random coils 24.87, 31.03, 17.89 and extended strands 16.24, 19.40, 17.89 (Table 2). The CURS proteins revealed the predominant nature of helix and coiling underlining the more compact and strongly bonded and transmembrane position of the CURS protein.

**Table 2:** Prediction of secondary structure of CURS proteins by SOPMA

Parameters	CURS1	CURS2	CURS3
α-Helix	47.72%	41.38%	44.74%
Extended β-strand	16.24%	19.40%	17.89%
Random coil	24.87%	31.03%	27.89%
Ambiguous state	0.00%	0.00%	0.00%

### Model validation

**Fig. 4:**
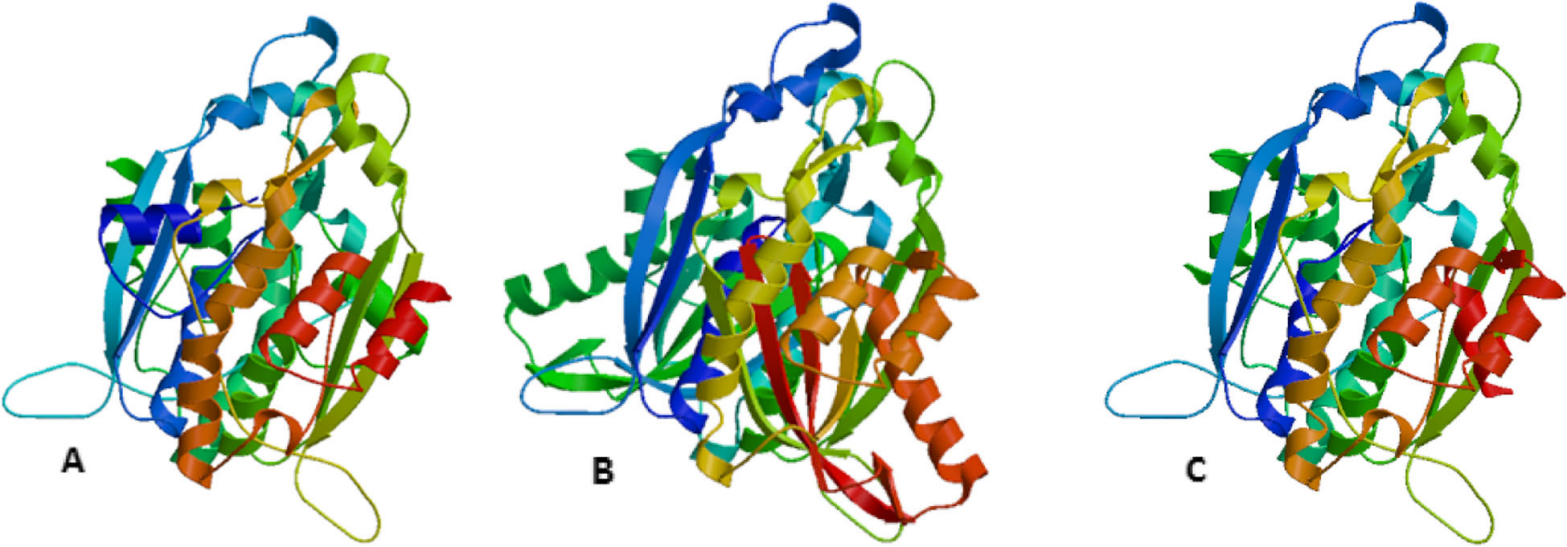
Model 3D structure of protein from *Curcuma longa***a** CURS1, **b** CURS2, and **c** CURS3

**Fig. 5:**
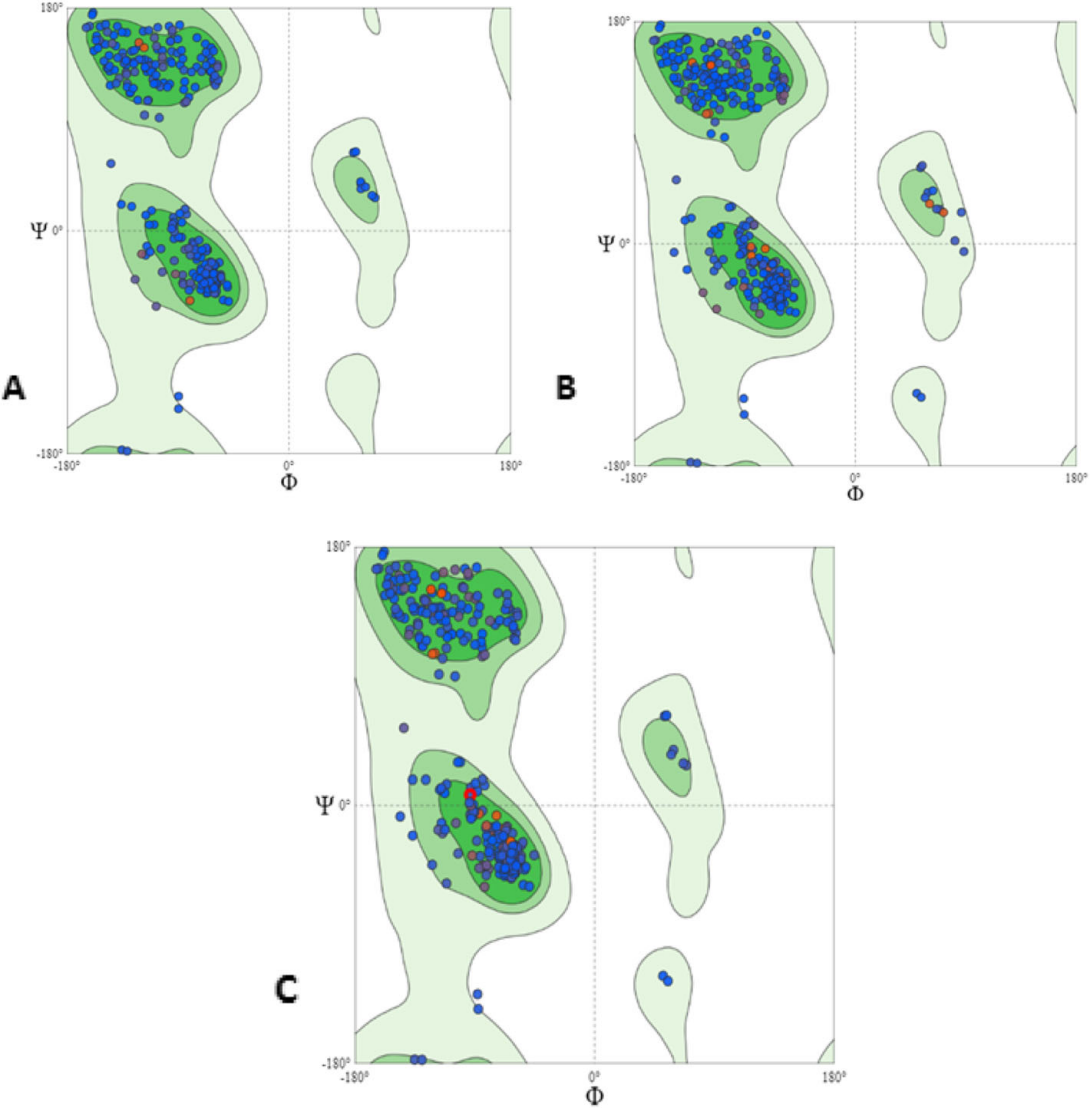
The stereochemical validation of the hypothetical model using Ramachandran plot of **a** CURS1, **b** CURS2, and **c** CURS3 proteins

Homology modeling of the CURS proteins was done using the automated homology protein modeling server of SWISS-MODEL, based on ProMod3, an open structure comparative modeling engine (Fig. 4a–c). The CURS1, CURS2, and CURS3 protein models were verified using the Ramachandran plot from the MolProbity program and validated all the amino acid residues of the modeled protein fit in the allowed regions of the Ramachandran plot. The CURS1 protein showed 1.3% MolProbity score, 97.67% residues were in the favored residues, 0% in the outliers regions; and the Clash score was 0.68%. The MolProbity score of CURS2 was 1.6%, favored residues were 95.45%, outliers regions with 0.22%; and Clash Score was 1.85%. In the CURS3 protein, the MolProbity score was 1.33%, 96.01% of the amino acids were in the favored regions, 0% in the outliers regions, and 0.52% Clash Score (Fig. 5a–c). The modeled proteins were submitted to PMDB and accession numbers were provided (PM0082212, PM0082213, and PM0082214).

**Fig. 6:**
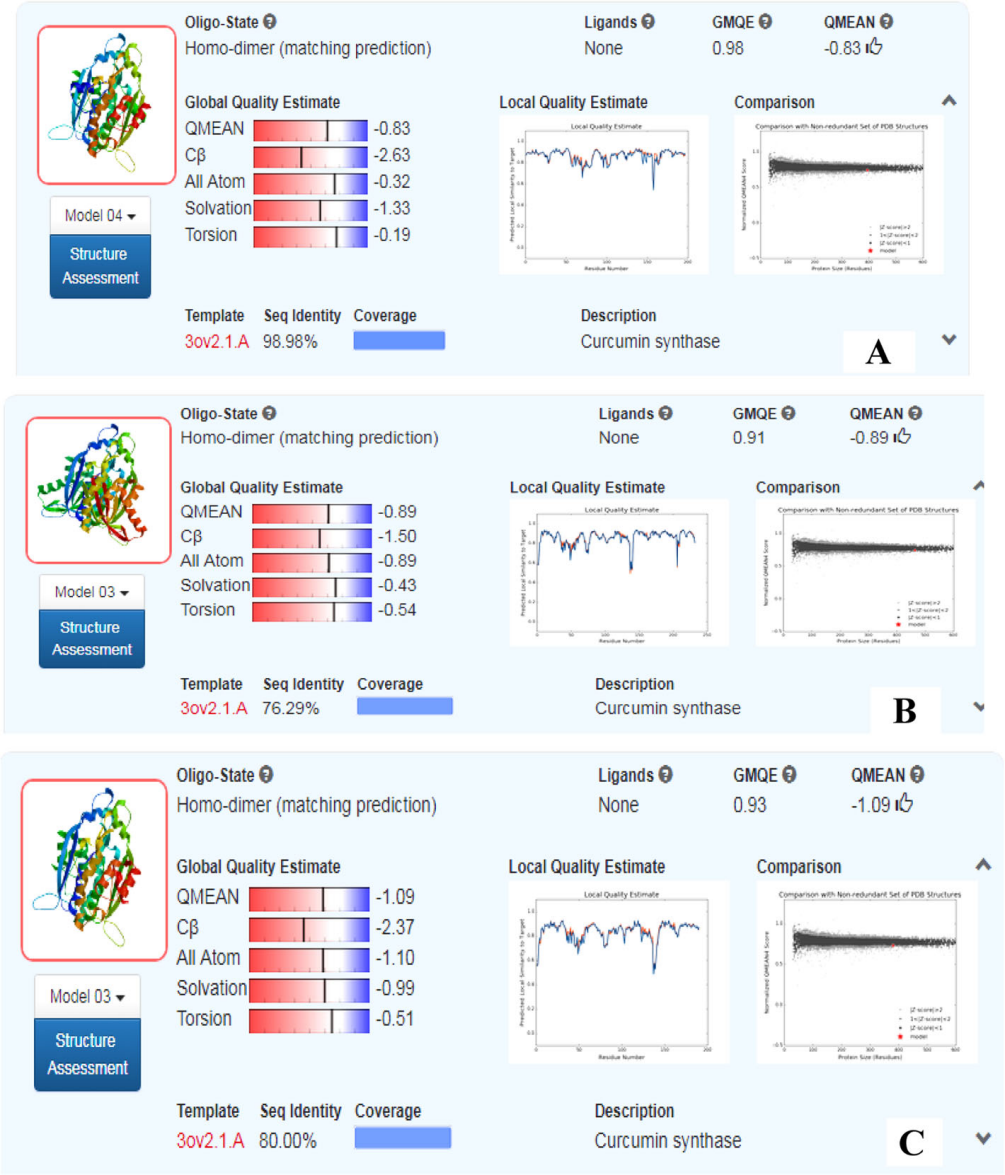
Quality estimation (GMQE, QMEAN, local quality estimate, and comparison plot) of **a** CURS1, **b** CURS2, and **c** CURS3 proteins

QMEAN Z-score was − 0.83, − 0.89, and − 1.09 for CURS1, CURS2, and CURS3, respectively. The individual Z-scores compared the interaction potential between Cβ atoms only. All atoms with the resolution potential and the torsion angle potential are shown in Fig. 6a–c. The “Local Quality” was estimated for each residue of the model (reported on the *x*-axis) and the expected similarity to the native structure (*y*-axis). Usually, residues showing a score below 0.6 are expected to be of low quality. In the “Comparison” plot (Fig. 6a–c), the model quality scores of individual models are related to scores obtained for experimental structures of similar size.

The QMEAN Z-score provided an estimate of the “degree of nativeness” of the structural features observed in the model on a global scale. It indicates whether the QMEAN score of the model is comparable to the expected score from experimental structures of similar size. QMEAN Z-score value of approximately zero specifies superior quality between the modeled structure and experimental structures. The obtained scores of − 4.0 or below indicate that the models with low quality. The QMEAN Z-scores of the CURS1, CURS2, and CURS3 proteins showed − 0.83, − 0.89, and − 1.09, respectively, and these results indicate that the proposed homology model is reliable and acceptable.

### Post-translational modifications

The process of post-translational modification mainly includes phosphorylation, glycosylation, ubiquitination, nitrosylation, methylation, acetylation, lipidation, and proteolysis. The CURS1 protein has 3 Ser, 2 Thr, and 1Tyr residues. S-146 has a score of 0.989 indicates its candidacy for a phosphorylation site than the other. CURS2 has 4 Ser, 5 Thr, and 2Tyr and S-196 has a score of 0.994, and CURS3 has 4 Ser, 1 Thr, and 3Tyr and S-33 with an overall score of 0.959.

## Discussion

The cloned putative sequences of CURS1, CURS2, and CURS3 showed better homology with the database CURS sequences and the ORF determination specified the protein characteristics. The aliphatic index of the protein is defined as the relative volume occupied by aliphatic side chains, which include alanine, valine, isoleucine, and leucine, and contribute to protein thermostability [20]. The predicted aliphatic index of CURS1 protein was 99.19%; CURS2, 89.30%; and CURS3, 86.37%. The isoelectric point is the condition where the amino acid maintains the same level of positive and negative charges and the net charge will be zero. Isoelectric points (pI) of CURS1, CURS2, and CURS3 were 4.93, 5.28, and 4.96 suggesting a moderately acidic nature of the protein. Approximately neutral pH is required in in vivo condition compared to in vitro for the optimum activity of the alkaline phosphatase enzyme [21]. The total number of positively charged and negatively charged residues refers to the total no. of lysine (K), arginine (R) and aspartate (D), and glutamate (E), respectively [22]. The instability indices were between 32.10, 37.84, and 31.33. The obtained instability indices for CURS1, CURS2, and CURS3 were lesser than 40, suggesting the stability of the proteins [23, 24]. GRAVY is used for the computational analysis of various physicochemical parameters for a given amino acid sequence [25]. Low range GRAVY value of 0.199, 1.118, and 0.058 indicates its high affinity for water that improves the solubility of a protein [25, 26].

Alpha helical structure is composed of methionine (M), alanine (A), leucine (L), glutamate (E), and lysine (K) amino acids, whereas the beta strand is composed of tryptophan (W), tyrosine (Y), phenylalanine (F), valine (V), isoleucine (I), and threonine (T); furthermore, glycine (G) and proline (P) amino acids help to build the relevant turns [23]. Such findings suggest that the numbers of amino acids are solely responsible for constructing the respective secondary structure of proteins. The percentage score of amino acid distribution infers that alpha helix is dominated over other secondary structures followed by the random coil, extended strand, and beta turn; Figs. 1, 2, and 3 represent the secondary structure of CURS proteins where the alpha helix is maximum than other structures.

The QMEAN quality estimations are based on different geometrical properties and provide both global (i.e., for the entire structure) and local (i.e., per residue) absolute quality estimates on the basis of one single model and its scoring function consists of a linear combination of six structural descriptors [27, 28]. The CASP experiment showed the optimization of weightage factors for the terms contributing to QMEAN has been performed on models from the seventh round of the (CASP7) [29]. QMEAN Z-scores are applied for the experimental structures from the PDB database [30]. The CURS proteins showed the highest phosphorylation sites, higher scores reflect the confidence of the prediction and similarity to one or more of the phosphorylation sites used in the method [31, 32]. Phosphorylation regulates innate inflammatory responses through the activation, cellular translation, and interaction of innate receptors, adaptors, and downstream signaling of molecules in response to infectious and dangerous signals [33].

## Conclusion

In the present study, bioinformatics tools were used to model the CURS (CURS1, CURS2, and CURS3) proteins of *Curcuma longa*. Multiple sequence alignment with CURS proteins had higher homologies with other CURS proteins. Primary structure analysis revealed that CURS proteins are acidic in nature and stable. The secondary structure analysis confirmed that in all three CURS proteins, the alpha helix dominated followed by random coil, extended strand, and beta turns. Tertiary structure predictions were analyzed by Swiss-model and the models were validated using PROCHECK’S Ramachandran plot. The models were validated and submitted in the PMDB server. Prediction of the 3D model of a protein by in silico analysis is a highly challenging aspect to confirm the data obtained from the NMR or X-ray crystallographic-based methods. Consequently, in silico analysis of protein structure is one of the very useful methods for studying the structural and functional aspects of the protein. Our results indicate that future studies with the quaternary structure of CURS proteins will provide a better insight into the exact or most probable molecular mechanisms involved in curcumin synthase. This report can throw light into the protein structure, physicochemical properties, structural motifs, and protein-protein interactions.

## Data Availability

Data sharing is not applicable to this article as no datasets were generated or analyzed during the current study.

## Abbreviations

cDNA: Complementary deoxyribonucleic acid
NCBI: National Centre for Biotechnology Information
NMR: Nuclear magnetic resonance
ORF: Open Reading Frame
PSIPRED: Position-specific iterative blast-based secondary structure prediction
RNA: Ribonucleic acid
SOPMA: Self-optimized prediction method with alignment
